# Inadequate prenatal care use and breastfeeding practices in Canada: a national survey of women

**DOI:** 10.1186/s12884-016-0889-9

**Published:** 2016-05-05

**Authors:** Christy Costanian, Alison K. Macpherson, Hala Tamim

**Affiliations:** School of Kinesiology & Health Science, Bethune College, York University, 4700 Keele Street, M3J 1P3 Toronto, ON Canada

**Keywords:** Breastfeeding practices, Women’s experiences, Prenatal Care Use, Canada

## Abstract

**Background:**

Previous studies have demonstrated that prenatal care (PNC) has an effect on women’s breastfeeding practices. This study aims to examine the influence of adequacy of PNC initiation and services use on breastfeeding practices in Canada.

**Methods:**

Data for this secondary analysis was drawn from the Maternity Experiences Survey (MES), a cross sectional, nationally representative study that investigated the peri-and post-natal experiences of mothers, aged 15 and above, with singleton live births between 2005 and 2006 in the Canadian provinces and territories. Adequacy of PNC initiation and services use were measured by the Adequacy of Prenatal Care Utilization Index. The main outcomes were mother’s intent to breastfeed, initiate breastfeeding, exclusively breastfeed, and terminate breastfeeding at 6 months. Multivariate logistic regression analysis assessed the adequacy of PNC initiation and service use on breastfeeding practices, while adjusting for socioeconomic, demographic, maternal, pregnancy and delivery related variables. Bootstrapping was performed to account for the complex sampling design.

**Results:**

Around 75.0 % of women intended to only breastfeed their child, with 90.0 % initiating breastfeeding, while 6 month termination and exclusive breastfeeding rates were at 52.0 % and 14.3 %, respectively. Regression analysis showed no association between adequate PNC initiation or services use, and any breastfeeding practice. Mothers with either a family doctor or a midwife as PNC provider were significantly more likely to have better breastfeeding practices compared to an obstetrician.

**Conclusions:**

In Canada, provider type impacts a mother’s breastfeeding decision and behavior rather than quantity and timing of PNC.

**Electronic supplementary material:**

The online version of this article (doi:10.1186/s12884-016-0889-9) contains supplementary material, which is available to authorized users.

## Background

Postpartum health behaviors such as initiation of breastfeeding and its maintenance for the first four to six months after birth have been shown to be among the low cost and effective interventions available for preventing neonatal morbidity and mortality across various settings [1, 2]. Exclusive breastfeeding, preceded by timely breastfeeding initiation and appropriate complementary feeding practices are universally accepted as essential elements for the satisfactory growth and development of infants and for prevention of childhood illness [3]. The World Health Organization (WHO) recommends that women exclusively breastfeed their infants till 6 months of age, and that they continue to breastfeed into the second year of life or longer. An infant is considered to be exclusively breastfed when he or she had received only breast milk with no other liquids (including water) or solids for the first 6 months of that infant’s age [4, 5]. Timely initiation of breastfeeding on the other hand, is defined as putting the newborn to the breast within one hour of birth with the most critical period of breastfeeding initiation being 0.5-2 h after birth [5]. Breastfeeding intention and initiation rates are high in Canada, at 90 % each, yet despite the documented short- and long-term medical and neurodevelopmental advantages of breastfeeding and its extensive promotion, the current rate of exclusive breastfeeding at 6 months after birth is low at 14.4 % [6, 7]. Previous studies conducted in Canada have identified factors that contribute to breastfeeding initiation, duration, and 6 month exclusive breastfeeding [6–13]. However, none investigated the role of prenatal care (PNC) utilization, in terms of both adequacy of services and timing of initiation on breastfeeding practices at the national scale.

Adequate PNC use during pregnancy has been shown to reduce maternal mortality and the risk of having adverse pregnancy and birth outcomes such as miscarriage, premature birth, low birth-weight, still birth and sudden unexpected death in infancy [14]. Variations in the recommendations of PNC timing, and number of visits exist [15–17], however, according to the Kotelchuk index based on American College of Obstetricians and Gynecologists’ (ACOG) recommendations, PNC is considered late and therefore inadequate, if commenced after the 4th month or if less than 50 % or more of the recommended number of prenatal care visits were made. Various psychosocial, maternal and hospital based factors play a role in establishing successful breastfeeding during the newborn period [18]. Previous studies conducted in developing countries have demonstrated that PNC has an effect on women’s breastfeeding practices and behaviors. For example, Nepalese women who had more than three visits to their PNC provider were 1.25 (95%CI = 1.02-1.54) times more likely to initiate breastfeeding within an hour after birth than women with one to three visits [19]. A cross-sectional study conducted in Tamil Nadu, South India, found that women who initiated PNC earlier, had more PNC visits and those who received information about breastfeeding during those visits were more likely to feed colostrum to their newborn [20]. Breastfeeding education and proper practices are introduced and implemented during prenatal care, with studies in Ethiopia [21] and Brazil [22] revealing that pregnant women who received prenatal guidance on breastfeeding were more likely to initiate breastfeeding. In fact, a study done by DiGirolamo et al. [23] using a national sample of pregnant women in the US, assessed the impact of the WHO’S Baby –Friendly practices initiative that included prenatal breastfeeding education, and showing the mother how to breastfeed within one hour of birth on breastfeeding duration. Results showed that receiving all practices in the initiative improved the chances of breastfeeding initiation and continuation [23]. Such studies show that PNC utilization has significant implications for breastfeeding practices such as, initiation, exclusivity and duration.

Although the prevalence rates of breastfeeding initiation and duration are known in Canada, the factors that influence women’s breastfeeding practices vis a vis inadequate prenatal care have yet to be addressed. To our knowledge, no studies in Canada have provided a direct link between both components of PNC adequacy and breastfeeding intent, initiation, duration, and exclusivity, while controlling for other covariates. One study in Quebec that assessed predictors of breastfeeding duration found a significant correlation between prenatal class attendance and exclusive breastfeeding [9]. Results of this study will help elucidate more effective ways of increasing antenatal service use, promoting healthy postpartum practices and therefore help lower the prevalence of adverse pregnancy outcomes as well as designing better breastfeeding support strategies. The present study, using data from a specialized survey on pre and post-delivery experiences among mothers residing in both the Canadian provinces and territories, aims to examine the utilization of prenatal care and its association with breastfeeding practices, such as intent, initiation, exclusive breastfeeding at 6 months, and breastfeeding termination at 6 months (i.e. any breastfeeding up till 6 months).

## Methods

### Study design and participants

Data from the Maternity Experiences Survey (MES) was used to determine the association between inadequate prenatal care in terms of initiation and services received and breastfeeding practices. This post-censual survey examined child births in the provinces and territories of Canada was sponsored by the Public Health Agency of Canada and conducted by Statistics Canada in 2006 [24]. The target population of the present study consisted of all women at least 15 years of age, who have given birth between February 15, 2006 and May 15, 2006 in the Canadian provinces and between November 1, 2005 and February 1, 2006 in the Canadian territories. These mothers must have had a single, live birth and must have been living with the baby at least one night per month in Canada. Mothers who lived on First Nation reserves and in collective residences were excluded. A stratified random sample of 8,545 Canadian women was selected, however, a total of 6,421 (78 %) women responded to the survey. Non-response to the survey was mainly from inability to establish contact with the mothers. Prior to data collection, an introductory letter and survey pamphlet were mailed to the women inviting them to participate in the survey [24].

### Data collection and measures

The data was collected through telephone interviews using a computer-assisted telephone interview application. Before starting the interviews, an introductory letter and pamphlet to the sample mothers were mailed. The letter introduced the survey and asked for their cooperation. In an attempt to recruit the highest number of mothers possible, a total of 25 calls per non-respondent case were made during different days of the week and different hours of the day. Interviews were conducted between the 5th and 14th month after delivery and lasted on average 45 min. The majority (96.9 %) of the interviews, however, were performed between the 5th and 9th month postpartum. The MES questionnaire was also available in 15 different languages. The MES project was presented to Health Canada’s Science Advisory Board; Health Canada’s Research Ethics Board and the Federal Privacy Commissioner and was approved by Statistics Canada’s Policy Committee. The MES has been previously described elsewhere [25].

### Outcome assessment

The primary outcomes were i) breastfeeding intention, measured by the following question “prior to giving birth, did you intend to feed by formula alone, breastfeeding alone or a combination of both?” ii) Breastfeeding initiation (yes/no) was assessed by the following question: “How long after the birth was the baby first put to the breast?” and defined as the infant’s first intake of breast milk. Women who responded that they had initiated the provision of milk directly from the breast after delivery were classified as “yes”. This definition is based on the WHO’s recommendation and was previously used by Forster et al. [26] and Geraghty and Rasmussen [27]. iii) Breastfeeding termination at 6 months was calculated using information about the infant’s age when breastfeeding was terminated and made dichotomous into < 6 months or ≥6 months. iv) Exclusive breastfeeding, based on the WHO definition, was calculated using information about breastfeeding termination and timing of introduction of liquids, semi-solid and solid foods and made dichotomous into <6 months or ≥6 months.

### Exposure assessment

The two separate dimensions of the Adequacy of Prenatal Care Utilization Index (APNCUI), namely adequacy of initiation (AI) and adequacy of services (AS) were the main exposures. AI characterizes the adequacy of the timing of PNC initiation, while AS characterizes the adequacy of the frequency of visits received during the time period after PNC is begun until delivery [28]. Specifically in the MES, AI was assessed by answering the following question: “How many weeks pregnant with baby were you when you had your first visit for prenatal care?” According to the ANPCUI, developed by Kotelchuck, PNC initiation is considered inadequate if routine care began after 17 weeks of pregnancy. Therefore, weeks 1–17 were combined and considered adequate, while weeks 18 and above were classified as inadequate PNC initiation [28, 29] AS on the other hand, was rated by answering this question “How many prenatal care visits did you have?” The APNCUI is designed to avoid incorrectly attributing underutilization of care to preterm birth by adjusting the required number of visits according to the gestational age at delivery. The expected number of visits is consistent with the ACOG’s guidelines for PNC use and is the ratio of actual visits to the recommended number of visits. *Inadequate care* required less than 50 % or more of the recommended number of visits (12 on average); *adequate* care required 50 to 110 % or more of the recommended number of visits [28, 29].

### Other variables

A wide range of covariates was used to investigate the characteristics of pregnant women’s breastfeeding practices in relation to inadequate PNC initiation and amount of services received were: i) socio-demographic factors including mother’s age at selected birth, urban–rural residence, immigration status, maternal level of education, and marital status; ii) maternal health characteristics including the mother’s previous depression diagnosis, and pre-pregnancy Body Mass Index (BMI) in kg/m^2^ (Underweight (<18.5), Normal (≥18.5 & <25),Overweight (≥25 & <30), Obese (≥30); iii) pregnancy-related factors including number of pregnancies including current pregnancy (gravidity), reaction to the pregnancy, health problems during pregnancy, smoking during pregnancy; iv) delivery characteristics including type of healthcare provider during prenatal care, type of delivery, birth setting; v) postpartum characteristics including baby’s NICU admission, work after pregnancy, support after pregnancy and intimate partner violence (IPV). All variables were directly self-reported by the mother.

### Statistical analysis

Proportions of breastfeeding intention, initiation, termination and exclusivity were estimated through population weights and examined across all the Canadian provinces. Applying the appropriate sample weights to the MES data allowed the survey data to be representative of the population. Please refer to Statistics Canada’s *Maternity Experiences Survey*, *2006*- *User guide* for more information: http://www23.statcan.gc.ca/imdb-bmdi/pub/document/5019_D1_T1_V1-eng.pdf. At the bivariate level, differences in the proportion of positively performing each breastfeeding practice were assessed among the different levels of each predictor using normalized weights. Chi square test was done to determine the differences in proportions of inadequate prenatal care initiation and service use, and socio-demographic, maternal health, pregnancy and delivery indicators among breastfeeding practices using normalized weights. The dependent variables for the logistic regression models were made dichotomous. The association between indicators of PNC use and each outcome variable (breastfeeding intention, initiation, termination, exclusivity) were analyzed using multivariable logistic regression. All known predictors of breastfeeding practices in the literature were considered for regression analysis. Adjusted odds ratios (ORs), and their 95 % confidence intervals (CIs) were estimated. To account for the complex sampling design, bootstrapping was performed to calculate the 95 % CI estimates. Population weights, normalized weights and bootstrap weights were all created by Statistics Canada and provided with the MES data file. All analyses, in exception to bootstrapping, were conducted using the Statistical Package for Social Sciences (SPSS, version 22.0). Bootstrapping was performed using Stata Data Analysis and Statistical Software (Stata, version 13.0). Statistical significance for all analyses was set at alpha <0.05 for a two-tailed test.

## Results

Analysis for the present study was restricted to 5,662 mothers with complete information on the questions required to calculate AI and AS (88.1 %), the main exposure variables. After excluding those with missing information on breastfeeding intention and initiation, a total of 5620 mothers weighted to represent 66,905 Canadian women, were included in the analysis for breastfeeding intention, while 5623 mothers weighted to represent 66,908 Canadian women were included in the breastfeeding initiation outcome. Samples for breastfeeding termination and exclusivity were limited to women who had babies aged ≥ 6 months at the time of the interview since this variable was based on the WHO’s definition of exclusive breastfeeding. Out of the 5662 mothers with complete data on the exposure variables, 4952 MES mothers had babies aged ≥ 6 months at the time of the interview. After exclusion of respondents with missing information on breastfeeding exclusivity and termination, 4820 women weighted to represent 57,351 Canadian women, and 4394 women weighted to represent 52,282 Canadian women, were considered in the analysis for the exclusive breastfeeding and breastfeeding termination outcomes, respectively.

Data showed that 74.8 % (95%CI = 72.4-76.2) of mothers intended to only breastfeed their infants before giving birth, with 90 % (95%CI = 88.9-90.4) initiating breastfeeding before hospital discharge. The 6 month exclusive breastfeeding rate was 14.3 % (95%CI = 13.4-15.2), with over 50 % (95%CI = 51.2-54.1) of mothers terminating breastfeeding at 6 months or more (results not shown). Unadjusted associations between each breastfeeding practice and AI and AS are shown in Additional file 1 Table S1 and Additional file 2 Table S2. Women who intended to breastfeed were significantly at a higher odds of having AS (OR = 1.19; 95%CI = 1.00-1.41). On the other hand, women who initiated PNC between 1 to 17 weeks of their pregnancy were almost twice as likely (OR = 1.74; 95%CI = 1.05-2.86) to initiate breastfeeding than women who initiated prenatal care use at 18 weeks or more of their pregnancy. Table 1 depicts the associations between adequacy of PNC initiation and services and each breastfeeding practice while controlling for other potential confounders. Neither AS nor AI, in the adjusted model, was associated with performing any breastfeeding practice. Mothers who had some postsecondary education or more, mothers with partners, who were pregnant for the first time, who did not smoke during their pregnancy, and whose PNC provider was either a family doctor or a midwife were at a significantly increased likelihood of intending to breastfeed prior to delivery, initiating breastfeeding, terminating breastfeeding at 6 months and achieving 6 month breastfeeding exclusivity. Also, mothers who did not have their babies admitted into the neonatal intensive care unit after birth were significantly more likely to intend to breastfeed and initiate breastfeeding, and terminate breastfeeding at 6 months and exclusively breastfeed.

**Table 1:** Adjusted association of breastfeeding intention, initiation, any breastfeeding at 6 months, and 6 month exclusive breastfeeding with inadequate prenatal care and other potential predictors

Independent Variables	Intended to Breastfeed	Initiated Breastfeeding	Terminated Breastfeeding at 6 months	Exclusive Breastfeeding at 6-months
	Adjusted OR (95%CI) ^a^	Adjusted OR (95%CI) ^a^	Adjusted OR (95%CI) ^a^	Adjusted OR (95%CI) ^a^
Adequacy of Services				
Adequate	1.19 (0.98-1.43)	1.07 (0.82-1.41)	1.08 (0.91-1.29)	1.07 (0.84 -1.37)
Inadequate	1.00	1.00	1.00	1.00
Adequacy of Initiation				
Weeks 1-17	1.19 (0.73-1.94)	1.47 (0.82-2.65)	0.99 (0.54-1.81)	0.95 (0.45-2.03)
Weeks 18 and above	1.00	1.00	1.00	1.00
*Maternal Demographics*				
Maternal age in years				
<20	1.00	1.00	1.00	1.00
20-39	0.99 (0.69-1.44)	1.34 (0.88-2.03)	2.03 (1.40-3.05)	1.38 (0.69-2.77)
> = 40	1.41 (0.76-2.53)	1.45 (0.65-3.28)	5.11 (2.68-9.77)	2.21 (0.92-5.34)
Urban–rural residence				
Rural area	1.00	1.00	1.00	1.00
Urban, population ≤499,999	1.06 (0.88-1.27)	1.10 (0.87-1.40)	1.03 (0.85-1.23)	0.93 (0.73-1.18)
Urban, population ≥500,000	1.09 (0.89-1.32)	1.37 (1.05-1.79)	1.24 (1.01-1.52)	0.98 (0.77-1.27)
Immigration to Canada				
No	1.27 (1.05-1.54)	0.60 (0.54-0.99)	0.76 (0.62-0.93)	0.82 (0.5-1.04)
Yes	1.00	1.00	1.00	1.00
Level of education				
High school or less	1.00	1.00	1.00	1.00
Some postsecondary education	1.51 (1.11-2.04)	2.41 (1.56-3.73)	1.33 (1.01-1.82)	1.94 (1.25-3.03)
University or College education	1.50 (1.25-1.78)	1.88 (1.50-2.37)	1.68 (1.40-2.02)	1.90 (1.40-2.53)
Marital Status				
No Partner	1.00	1.00	1.00	1.00
Partner	1.28 (1.02-1.64)	1.23 (1.01-1.38)	1.48 (1.04-1.38)	1.70 (1.05-2.07)
*Maternal Health Characteristics*				
Previous depression diagnosis				
No	0.98 (0.82-1.19)	0.91 (0.70-1.17)	0.99 (0.81-1.2)	0.94 (0.870-1.23)
Yes	1.00	1.00	1.00	1.00
Pre-pregnancy BMI (kg/m^2^)				
Underweight	1.13 (0.82-1.47)	1.21 (1.00-1.47)	1.40 (1.02-1.47)	1.37 (1.01-2.12)
Normal	1.21 (0.87-1.67)	1.73 (1.03-1.87)	1.50 (1.3-1.75)	1.24 (1.02-1.51)
Overweight or Obese	1.00	1.00	1.00	1.00
*Pregnancy*-*Related Characteristics*				
Gravidity				
Primigravida	1.54 (1.33-1.79)	1.64 (1.33-2.03)	1.18 (1.04-2.56)	1.19 (1.02-2.68)
Multigravida	1.00	1.00	1.00	1.00
Reaction to pregnancy				
Happy	1.08 (0.64-1.82)	0.78 (0.38-1.58)	0.87 (0.51-1.50)	0.85 (0.49-1.47)
Indifferent	0.92 (0.62-1.37)	0.76 (0.43-1.32)	1.12 (0.73-1.73)	0.95 (0.47-1.93)
Unhappy	1.00	1.00	1.00	1.00
Health problems during pregnancy				
No	1.16 (0.98-1.37)	1.22 (0.98-1.52)	1.08 (0.93-1.28)	1.14 (0.91-1.42)
Yes	1.00	1.00	1.00	1.00
Cigarette smoking during pregnancy				
No	2.07 (1.66-2.58)	2.53 (1.94-3.30)	2.52 (1.95-3.26)	2.76 (1.71-4.44)
Yes	1.00	1.00	1.00	1.00
*Delivery Characteristics*				
Type of PNC provider				
Obs/Gyn	1.00	1.00	1.00	1.00
Family Doctor	1.20 (1.04-1.39)	1.47 (1.21-1.80)	1.31 (1.12-1.54)	1.26 (1.02-1.54)
Midwife	2.60 (1.69-4.03)	3.60 (1.61-7.87)	2.28 (1.57-3.32)	1.91 (1.31-2.78)
Nurse or nurse practitioner/other	1.10 (0.71-2.52)	1.10 (0.71-2.52)	1.16 (0.71-2.52)	0.99 (0.71-2.52)
Type of Delivery				
Vaginal	1.27 (1.09-1.47)	1.14 (0.92-1.42)	1.18 (1.00-1.39)	1.13 (0.91-1.40)
Cesarean	1.00	1.00	1.00	1.00
Birth Setting				
Hospital	1.00	1.00	1.00	1.00
Birthing center/Private home	1.44 (0.94-11.23)	2.35 (0.91-12.88)	2.83 (1.17-6.83)	2.41 (1.34-4.03)
*Postpartum Characteristics*				
Baby’s admission to the NICU				
No	1.23 (1.03-1.47)	1.67 (1.31-2.13)	1.59 (1.30-1.95)	1.32 (1.30-1.95)
Yes	1.00	1.00	1.00	1.00
Work after delivery				
No	1.13 (0.94-1.37)	1.29 (0.99-1.66)	1.30 (1.07-1.58)	1.40 (1.07-1.58)
Yes	1.00	1.00	1.00	1.00
Support After delivery				
All of the time/Most of the time	1.02 (0.73-1.40)	1.09 (0.70-1.70)	0.88 (0.60-1.32)	0.62 (0.37-1.02)
Some of the time	0.74 (0.51-1.08)	0.99 (0.58-1.67)	0.99 (0.64-1.24)	0.69 (0.46-1.05)
None/Little of the time	1.00	1.00	1.00	1.00
Intimate Partner Violence				
No	0.83 (0.66-1.04)	0.72 (0.52-1.01)	1.15 (0.92-1.45)	1.23 (0.86-1.77)
Yes	1.00	1.00	1.00	1.00

^a^ CI-Confidence Interval

## Discussion

This study aimed to investigate the impact of two indicators of PNC service use (initiation and services), on breastfeeding practices, in terms of intention, initiation, termination and exclusivity among mothers throughout the Canadian provinces and territories. Results revealed that neither AS nor AI was associated with an increased likelihood of performing any breastfeeding practice. This study also suggests that women whose PNC provider was a either a family doctor or a midwife were more likely to intend to breastfeed, initiate breastfeeding, breastfeed exclusively, and maintain breastfeeding for 6 months. Health care practitioners and other allied health personnel have a critical role in serving as advocates and supporters of successful breastfeeding and future studies are needed to better elucidate how to effectively provide support for mothers in the postpartum period.

Although breastfeeding intention before birth and initiation were high at 75 %, and 90 % respectively, 6 month termination and exclusive breastfeeding rates were lower at 52 % and 14.3 %, respectively. These breastfeeding prevalence rates were similar to that found in developed countries. For example, three quarters of women in the US initiate breastfeeding, but only 35 % exclusively breastfeed through 3 months [30]. The low estimate obtained for exclusive breastfeeding is similar to other developed countries. In fact, data from 24 developed countries showed that rates ranged from 13 % to 96 % at 3 months, from 7 % to 63 % at 4 months and from 3 % to 44 % at 6 months of exclusive breastfeeding [31]. Given that breastfeeding has a dose–response effect, with increased benefits being proportionate to the extent of exclusive breastfeeding and duration of breastfeeding [32], this suboptimal breastfeeding rate found in this study might indicate that mothers and their infants are not receiving the maximum health benefits breastfeeding provides.

Results of the multivariable logistic regression showed no association between a mother’s intent to breastfeed, initiate breastfeeding, exclusively breastfeed, and terminate breastfeeding at 6 months and AS or AI. These results were contradictory to Nwaru et al. [33] who found that having more than three prenatal care visits, having sufficient tests and advice were positively associated with breastfeeding initiation an hour after birth among women aged 15 to 49 in the Nepalese Demographic and Health Survey (NDHS). Another study in Brazil reported that mothers who received prenatal guidance regarding the advantages of breastfeeding were more likely to initiate breastfeeding within the first hour of birth [22]. This finding was nonetheless consistent with the a Cochrane systematic review that found no difference in breastfeeding practice between women who had a lactation consultation and received a booklet on breastfeeding during their PNC visits versus those who did not have these extra services during PNC [34]. Regardless of the quantity and timing of PNC service use, PNC remains an opportunity for education on breastfeeding and its potential health benefits. Although the content of PNC varies across providers, it generally focuses on conducting health assessments, screening for potential complications and provision of nutritional and health information on various aspects related to the pregnancy. Although the frequency and the timing of prenatal care are important to ensure a healthy pregnancy, the content of prenatal (sufficient test and advice) during pregnancy should also be taken into account [33]. If breastfeeding initiation is delayed, then the infant may be deprived from the benefit of colostrum [35]. Benefits for breastfeeding mothers include a reduced risk of osteoporosis, a faster recovery from the pregnancy, a reduced risk of ovarian cancer, and an increased attachment of mother and baby [36].

A myriad of socio demographic, health – related and personal factors could affect a mother’s decision to breastfeed, however previous studies have identified the infant’s fathers’ and health professionals’ opinions as significant determinants of women’s breastfeeding outcomes. Our findings revealed that women who sought PNC from a family doctor or a midwife more likely to perform good breastfeeding practices. Using data from the Infant Feeding Practices Study II, Kornides and Kitsantas [37] found that the prenatal clinician’s opinion about breastfeeding seemed to have the strongest impact on breastfeeding initiation; if the woman’s clinician supported breastfeeding only, then a woman was almost two times more likely to initiate breastfeeding compared to those women whose clinician encouraged the use of formula only or both formula and breastfeeding. Odom et al. [38] found similar results whereby health provider’s advice on breastfeeding played a major role in influencing mothers’ practices. Routine PNC being delivered by non obs/gyn providers in the US has seen an increase in recent years [39]; the reason for this might lie in the convergence of knowledge of routine practices among practitioners. Nonetheless, breastfeeding education and/or support increases breastfeeding duration rates and decreases no breastfeeding rates at birth, <1 month and 1–5 months, as a systematic review conducted by Haroon et al. [40] has demonstrated. In fact, women in our study who were with a partner were more likely to have good breastfeeding behaviour. The role of the spouse could be to provide social support for the mother, which may facilitate the decision and the process of breastfeeding. Indeed, fathers do play a role in breastfeeding decisions. Odom et al. [38], using data from the Infant Feeding Practices Study II, examined how the opinions of individuals in a woman’s support network influence her decision to breastfeed. They found that never breastfeeding was significantly associated with the infant’s father preferring only formula feeding.

Prenatal breastfeeding intention is a strong indicator of breastfeeding initiation and duration [41], and so breastfeeding practices might have common predictors. Several other factors were associated with all four breastfeeding practices examined in this study, after controlling for all other confounding variables. Our results showed that maternal education was a strong factor in predicting breastfeeding intent, initiation, exclusivity, and duration. This was consistent with previous findings of studies done in Southern California [42], and Nepal [43], but not in Australia [44]. Our results found that primigravidas (pregnant for the first time) were more likely to have positive breastfeeding habits. Previous studies have examined the relationship between breastfeeding practices and parity (number of children), which can be a proxy for gravidity. Previous research is contradictory with some studies finding a positive association between both initiation and duration of breastfeeding and parity [8], whereas others not observing any association once confounders were controlled for [45]. Interestingly, previous breastfeeding experience has been shown to play a more significant role than parity in subsequent breastfeeding behavior. Results from a prospective cohort study in Hong Kong indicated that longer prior breastfeeding experience positively influenced subsequent breastfeeding duration. However, participants with longer previous breastfeeding durations tended to have shorter current breastfeeding durations [46]. An explanation to the results we obtained might be that first time expectant mothers might be more enthusiastic to take care of their newborn, and so may be more likely to adhere to proper breastfeeding practices. In this study, non-smoking women were more likely to adhere to good breastfeeding practices than women who smoked. This finding was consistent with a Pregnancy Related Assessment and Monitoring System survey among 1789 Missouri mothers which found that smokers initiated breastfeeding less often and weaned earlier than non-smokers [47]. These results could be explained by previous studies that have shown use of fewer health care services in general and PNC in particular among smokers [48].

Data for this study considered all the Canadian provinces, resulting in a representative picture of the population, enabling the generalizability of our results. This study also utilized a large sample size allowing for ample statistical power, with population weights accounting for nonresponse. In addition, confounding bias was minimized due to the variety of potential predictors that were controlled for in the analysis. Lastly, this is the first nationwide study that examined the two dimensions of inadequate PNC use and other factors correlated with breastfeeding practices using a more accurate and comprehensive set of measures of PNC utilization [49]. However, a few caveats do exist. Incomplete information on some exposure and outcome variables in this study might lead to potential selection bias. In addition, the cross sectional nature of the study design does not allow us to infer causality. Exposures and outcomes were self- reported, thereby introducing potential misclassification bias. Moreover, since interviews were conducted 5–9 months post-delivery, recall bias of the outcome variables may have been introduced. Yet the magnitude of recall bias on most of our outcome variables is likely to be minimal as a review on the validity and reliability of maternal recall of breastfeeding practices found that mothers’ recall will provide an accurate estimate of initiation and duration of any breastfeeding, especially when the duration is recalled after a short period of time (≤3 years) [50]. Although the APNCUI, is an accurate and widely used tool to measure PNC adequacy, it only assesses its utilization and not the delivered quality. It is also worthwhile to mention that the time lapse between data collection for this study and this analysis might render the results to be less relevant to the current context given the changes in health services and perinatal care education over this period.

## Conclusions

In conclusion, PNC is not only important to ensure a healthy pregnancy for both a woman and her baby; the broad goal of modern care is to promote the health of the mother, child, and family through the pregnancy, delivery, and the child’s development. The present study‘s results suggest that it is not the quantity or timing of PNC that determines healthy breastfeeding decision and behavior, but rather the PNC provider type that is associated with breastfeeding intention, initiation, exclusivity, and 6 month termination rates. During the prenatal period, health care providers of any form, whether specialized, general or allied, have an opportunity to communicate the importance of breastfeeding and its benefits to a mother and her baby.

### Ethics approval and consent to participate

An ethical review is not a requirement since many universities, including York University decided that secondary analysis using Statistics Canada data, does not require an ethics review. Also, since access to data is contingent upon security clearance by Statistics Canada, and becoming a deemed employee of Statistics Canada. As a deemed employee of Statistics Canada, the lead author has the right to access the data specified in the contract under the Statistics Act. For more information, please see the following: http://www.statcan.gc.ca/eng/rdc/faq#a11a.

### Consent for publication

Not applicable.

### Availability of data and materials

Data will not be shared, as it is not the author’s property, but belongs to Statistics Canada, and so data cannot be release after the termination of the project deadline as per the contract signed between the authors and Statistics Canada.

## Additional files

Additional file 1: Table S1. Bivariate analysis and unadjusted association of breastfeeding intention, initiation, 6-month exclusive breastfeeding and breastfeeding termination at 6 months with inadequate prenatal care and other potential predictors. (PDF 1505 kb) Additional file 2: Table S2. Adjusted association of breastfeeding intention, initiation, any breastfeeding at 6 months, and 6 month exclusive breastfeeding with inadequate prenatal care and other potential predictors. (PDF 1505 kb)

## Abbreviations

ACOG: American College of obstetricians and gynecologists
AI: adequacy of initiation
APNCUI: adequacy of prenatal care utilization index
AS: adequacy of services
MES: maternity experiences survey
NDHS: Nepalese demographic and health survey
PNC: prenatal care
WHO: world health organization
