# Supplementary material for: Inadequate prenatal care use and breastfeeding practices in Canada: a national survey of women
Source: BMC Pregnancy Childbirth. 2016 May 5;16:100. doi: 10.1186/s12884-016-0889-9 (PMC4858884; doi:10.1186/s12884-016-0889-9)
Supplement: Additional file 1: Table S1. — Bivariate analysis and unadjusted association of breastfeeding intention, initiation, 6-month exclusive breastfeeding and breastfeeding termination at 6 months with inadequate prenatal care and other potential predictors. (PDF 1505 kb) [file 12884_2016_889_MOESM1_ESM.pdf]

Table 1: Bivariate analysis and unadjusted association of breastfeeding intention, initiation, 6-month exclusive breastfeeding and breastfeeding termination at 6 months with inadequate prenatal care and other potential predictors

Independent Variables	Intended to Breastfeed (N=5620 [87.5%]¥)		Initiated Breastfeeding (N=5623 [87.6%]¥)		Terminated Breastfeeding at 6 months (N=4394 [68.4%]¥)		Exclusive Breastfeeding at 6-months (N=4820 [75.0%]¥)	
	**N (%)***	**Unadjusted OR (95%CI)†**	**N (%)***	**Unadjusted OR (95%CI)†**	**N (%)***	**Unadjusted OR (95%CI)†**	**N (%)***	**Unadjusted OR (95%CI)†**
**Adequacy of Services**								
Adequate	3547 (76.4)	**1.19 (1.00-1.41)**	4176(89.9)	1.03 (0.78-1.26)	2183(60.4)	1.02 (0.87-1.20)	597 (15.0)	0.99 (0.80-1.24)
Inadequate	712 (73.0)	1.00	877(89.9)	1.00	465 (59.7)	1.00	128 (15.0)	1.00
**Adequacy of Initiation**								
Weeks 1-17	4184 (75.9)	1.27 (0.81-1.99)	4964(90.0)	**1.74 (1.05-2.86)**	2597(60.2)	1.93 (0.57-1.50)	711 (15.1)	1.06 (0.53-2.10)
Weeks 18 and above	76 (71.2)	1.00	88(83.8)	1.00	51 (62.0)	1.00	14.1 (14.3)	1.00
Maternal Demographics								
**Maternal age in years**								
<20	116 (72.0)	1.00	133(83.4)	1.00	33(26.5)	1.00	8 (5.3)	1.00
20-39	4012 (76.0)	1.22 (0.91-1.65)	4757(90.0)	**1.80 (1.28-2.50)**	2513(60.8)	**4.30(3.05-6.03)**	688 (15.2)	**3.20(1.69-6.07)**
>=40	124 (76.0)	1.23(0.76-1.98)	149(90.8)	**1.96 (1.00-3.83)**	96 (77.6)	**9.56 (5.45-11.75)**	30 (22.3)	**5.12 (2.33-11.23)**
**Urban-rural residence**								
Rural area	735 (74.7)	1.00	854(87.0)	1.00	423 (56.3)	1.00	121 (14.3)	1.00
Urban, population ≤499,999	1525 (75.7)	1.05(0.88-1.25)	1770(87.8)	1.09 (0.87-1.36)	855 (56.2)	0.99 (0.83-1.18)	229 (13.5)	0.93 (0.73-1.17)
Urban, population ≥500,000	1874 (76.7)	1.11(0.93-1.33)	2262(92.6)	**1.88 (1.48-2.40)**	1288 (65.3)	**1.45 (1.22-1.73)**	350 (16.5)	1.18 (0.93-1.48)
**Immigration to Canada**								
No	3366 (76.5)	1.18 (1.00-1.39)	3870(87.4)	0.73(0.60-1.12)	1924 (57.4)	0.60 (0.49-0.70)	527 (14.0)	0.72 (0.59-0.88)
Yes	878 (73.3)	1.00	1161(96.9)	1.00	712 (69.6)	1.00	193 (18.4)	1.00
**Level of education**								
High school or less	762 (67.2)	1.00	935 (82.4)	1.00	373 (44.8)	1.00	73 (7.6)	1.00
Some postsecondary education	257 (75.8)	**1.53 (1.16-2.01)**	311(91.3)	**2.24 (1.50-3.35)**	147 (54.6)	**1.49 (1.13-1.96)**	46 (15.4)	**2.21 (1.45-3.37)**
University or College education	3207 (78.3)	**1.76 (1.50-2.04)**	3760(91.7)	**2.36 (1.95-2.85)**	2097 (64.6)	**2.25 (1.92-2.62)**	599 (17.1)	**2.50 (1.91-3.27)**
**Marital Status**								
No Partner	317 (68.2)	1.00	402 (86.6)	1.00	140 (40.0)	1.00	27 (6.7)	1.00
Partner	3932 (75.9)	**1.53 (1.24-1.88)**	4633(90.2)	1.41 (1.09-1.84)	2500 (62.2)	**2.52 (2.01-3.16)**	697 (15.8)	**2.64 (1.74-3.99)**
Maternal Health Characteristics								
**Previous depression diagnosis**								
No	3604 (76.2)	1.13 (0.96-1.33)	4262(90.1)	1.19 (0.94-1.50)	2267 (61.3)	**1.31 (1.10-1.54)**	618 (15.3)	1.13 (0.88-1.43)
Yes	651 (73.4)	1.00	781 (88.5)	1.00	377 (54.8)	1.00	105 (13.8)	1.00
**Pre-pregnancy BMI (kg/m^2)**								
Underweight	247 (75.5)	1.10(0.82-1.47)	304(92.9)	**1.38 (1.14-1.64)**	158 (59.2)	1.27 (0.96-1.68)	45 (15.9)	1.33 (0.89-1.97)
Normal	2539 (77.1)	1.21(1.05-1.39)	2989(90.8)	**1.82 (1.14-2.91)**	1674 (64.2)	**1.56 (1.36-1.80)**	460 (16.2)	**1.36 (1.13-1.63)**
Overweight or Obese	1405(73.7)	1.00	1675(87.7)	1.00	775 (53.4)	1.00	201 (12.4)	1.00
Pregnancy-Related Characteristics								
**Gravidity**								
Primigravida	1558 (81.2)	**1.57 (1.40-1.80)**	1786(92.6)	**1.63 (1.34-1.97)**	880 (57.0)	0.81(0.72-0.92)	220 (13.3)	0.81(0.67-0.97)
Multigravida	2695 (73.2)	1.00	3252(88.4)	1.00	1760 (62.0)	1.00	503 (16.0)	1.00

* Percentage of each breastfeeding practice with the characteristic in the row. †CI-Confidence Interval; ¥: Includes all women with complete information on exposures and outcome.

Table 1: Bivariate analysis and unadjusted association of breastfeeding intention, initiation, 6-month exclusive breastfeeding and breastfeeding termination at 6 months with inadequate prenatal care and other potential predictors (Cont'd)

**Independent Variables**	**Intended to Breastfeed**		**Initiated Breastfeeding**		**Terminated Breastfeeding at 6 months**		**Exclusive Breastfeeding at 6-months**	
	**N (%)***	**Unadjusted OR (95%CI)†**	**N (%)***	**Unadjusted OR (95%CI)†**	**N (%)***	**Unadjusted OR (95%CI)†**	**N (%)***	**Unadjusted OR(95%CI)†**
**Reaction to pregnancy**								
Happy	3967(76.0)	1.25 (0.88-1.77)	4697(89.9)	0.84 (0.44-1.57)	2493(61.1)	1.40 (0.97-2.02)	675(15.1)	1.02 (0.61-1.73)
Indifferent	164(73.4)	1.09 (0.68-1.75)	198 (88.6)	0.96 (0.58-1.56)	81 (46.1)	0.76 (0.48-1.22)	26 (13.3)	0.88 (0.45-1.75)
Unhappy	115(72.0)	1.00	145(90.3)	1.00	68 (52.9)	1.00	21 (14.8)	1.00
**Health problems during pregnancy**	3249(76. 5)	**1.17 (1.01-1.36)**	3847(90.6)	**1.37 (1.12-1.66)**	2054(61.4)	**1.20 (1.03-1.40)**	574 (15.7)	**1.25 (1.02-1.53)**
No	1006(73.5)	1.00	1200(87.6)	1.00	595 (56.9)	1.00	151 (13.0)	1.00
Yes								
**Cigarette smoking during pregnancy**								
No	3904(77.5)	**2.14 (1.77-2.58)**	4610(91.5)	3.49 (2.80-4.33)	2520(63.0)	**3.52 (2.80-4.42)**	699 (16.2)	**3.57 (2.32-5.50)**
Yes	351(61.6)	1.00	435 (75.6)	1.00	126 (32.6)	1.00	26 (5.14)	1.00
Delivery Characteristics								
**Type of PNC provider**								
Obs/Gyn	2388(73.6)	1.00	2885(88.9)	1.00	1457(58.0)	1.00	375 (13.5)	1.00
Family Doctor	1500(76.8)	**1.19 (1.05-1.36)**	1766(90.3)	1.16 (0.97-1.4)	929 (60.7)	1.12 (0.98-1.28)	253 (15.0)	1.13 (0.94-1.36)
Midwife	317(90.0)	**3.22(2.20-4.70)**	340 (96.8)	**3.72 (1.87-7.41)**	233(7.5)	**2.82 (2.07-3.84)**	90 (29.5)	**2.67 (1.98-3.60)**
Nurse or other	38(75.5)	1.10(0.63-1.95)	42(86.2)	0.78 (0.49-0.38)	21 (53.4)	0.83(0.44-1.54)	3 (7.90)	0.55 (0.20-1.49)
**Type of Delivery**								
Vaginal	3209(77.0)	**1.26 (1.09-1.43)**	3768(90.3)	1.17 (0.97-1.43)	2023(61.6)	**1.24 (1.08-1.43)**	566 (15.7)	**1.25 (1.02-1.53)**
Cesarean	1051(72.6)	1.00	1284(88.7)	1.00	626 (56.4)	1.00	159 (13.0)	1.00
**Birth Setting**								
Hospital	4149 (75.5)	1.00	4833(89.9)	1.00	2560(59.7)	1.00	682 (14.5)	1.00
Birthing center/Private home	106 (89.2)	**2.67 (1.32-5.42)**	112(87.4)	3.61 (0.98-10.9)	87(87.6)	**4.80 (2.39-9.65)**	41 (39.7)	**4.80 (2.39-9.65)**
Postpartum Characteristics								
**Baby's admission to the NICU**								
No	3739(76.3)	**1.23 (1.03-1.47)**	4440(90.6)	**1.77 (1.42 -2.21)**	2383(62.0)	**1.71 (1.42-2.06)**	661 (15.7)	**1.61 (1.21-2.14)**
Yes	72(72.3)	1.00	603 (84.5)	1.00	264 (49.0)	1.00	63 (10.4)	1.00
**Work after delivery**								
No	3678(76.0)	1.06 (0.88-1.26)	4366(90.2)	**1.32 (1.05-1.66)**	2301(61.2)	**1.30 (1.07-1.55)**	642 (15.6)	**1.40 (1.09-1.80)**
Yes	572(75.0)	1.00	670 (87.5)	1.00	340 (54.9)	1.00	81 (11.7)	1.00
**Support after delivery**								
All of the time /Most of the time	3642(76.7)	1.18 (0.89-1.57)	4274(89.9)	1.17 (0.80-1.70)	2228(60.0)	0.89 (0.67-1.18)	602 (14.8)	0.70 (0.45-1.09)
Some of the time	384 (69.2)	0.80 (0.58-1.12)	499 (90.2)	1.21 (0.75-1.93)	273 (61.4)	0.94 (0.66-1.34)	71 (14.8)	0.69 (0.49-1.00)
None /Little of the time	228 (73.5)	1.00	274 (88.4)	1.00	147 (62.7)	1.00	52 (19.9)	1.00
**Intimate Partner Violence**								
No	3799 (76.0)	1.04 (0.86-1.27)	4499(90.0)	1.11(0.86-1.46)	2411(61.7)	**1.66 (1.36-2.01)**	669 (15.6)	**1.54 (1.13-2.09)**
Yes	450 (75.2)	1.00	5032(89.9)	1.00	231 (49.3)	1.00	56 (10.7)	1.00

* Percentage of each breastfeeding practice with the characteristic in the row. †CI-Confidence Interval
