# Supplementary material for: Inadequate prenatal care use and breastfeeding practices in Canada: a national survey of women
Source: BMC Pregnancy Childbirth. 2016 May 5;16:100. doi: 10.1186/s12884-016-0889-9 (PMC4858884; doi:10.1186/s12884-016-0889-9)
Supplement: Additional file 2: Table S2. — Adjusted association of breastfeeding intention, initiation, any breastfeeding at 6 months, and 6 month exclusive breastfeeding with inadequate prenatal care and other potential predictors. (PDF 1505 kb) [file 12884_2016_889_MOESM2_ESM.pdf]

Table 2: Adjusted association of breastfeeding intention, initiation, any breastfeeding at 6 months, and 6 month exclusive breastfeeding with inadequate prenatal care and other potential predictors

Independent Variables	Intended to Breastfeed	Initiated Breastfeeding	Terminated Breastfeeding at 6 months	Exclusive Breastfeeding at 6-months
	Adjusted OR(95%CI)†	Adjusted OR (95%CI)†	Adjusted OR (95%CI)†	Adjusted OR (95%CI)†
**Adequacy of Services**				
Adequate	1.19 (0.98-1.43)	1.07 (0.82-1.41)	1.08 (0.91-1.29)	1.07 (0.84 -1.37)
Inadequate	1.00	1.00	1.00	1.00
**Adequacy of Initiation**				
Weeks 1-17	1.19 (0.73-1.94)	1.47 (0.82-2.65)	0.99 (0.54-1.81)	0.95 (0.45-2.03)
Weeks 18 and above	1.00	1.00	1.00	1.00
Maternal Demographics				
**Maternal age in years**				
<20	1.00	1.00	1.00	1.00
20-39	0.99 (0.69-1.44)	1.34 (0.88-2.03)	**2.03 (1.40-3.05)**	1.38 (0.69-2.77)
>=40	1.41(0.76-2.53)	1.45(0.65-3.28)	**5.11 (2.68-9.77)**	2.21 (0.92-5.34)
**Urban-rural residence**				
Rural area	1.00	1.00	1.00	1.00
Urban, population ≤499,999	1.06(0.88-1.27)	1.10 (0.87-1.40)	1.03 (0.85-1.23)	0.93 (0.73-1.18)
Urban, population ≥500,000	1.09(0.89-1.32)	1.37 (1.05-1.79)	**1.24 (1.01-1.52)**	0.98 (0.77-1.27)
**Immigration to Canada**				
No	**1.27 (1.05-1.54)**	**0.60 (0.54-0.99)**	**0.76 (0.62-0.93)**	0.82 (0.5-1.04)
Yes	1.00	1.00	1.00	1.00
**Level of education**				
High school or less	1.00	1.00	1.00	1.00
Some postsecondary education	**1.51 (1.11-2.04)**	**2.41 (1.56-3.73)**	**1.33 (1.01-1.82)**	**1.94 (1.25-3.03)**
University or College education	**1.50 (1.25-1.78)**	**1.88 (1.50-2.37)**	**1.68 (1.40-2.02)**	**1.90 (1.40-2.53)**
**Marital Status**				
No Partner	1.00	1.00	1.00	1.00
Partner	**1.28 (1.02-1.64)**	**1.23 (1.01-1.38)**	**1.48 (1.04-1.38)**	**1.70 (1.05-2.07)**
Maternal Health Characteristics				
**Previous depression diagnosis**				
No	0.98 (0.82-1.19)	0.91 (0.70-1.17)	0.99 (0.81-1.2)	0.94 (0.870-1.23)
Yes	1.00	1.00	1.00	1.00
**Pre-pregnancy BMI (kg/m^2)**				
Underweight	1.13(0.82-1.47)	**1.21(1.00-1.47)**	**1.40 (1.02-1.47)**	**1.37 (1.01-2.12)**
Normal	1.21(0.87-1.67)	**1.73(1.03-1.87)**	**1.50 (1.3-1.75)**	**1.24 (1.02-1.51)**
Overweight or Obese	1.00	1.00	1.00	1.00

†CI-Confidence Interval

Table 2: Adjusted association of breastfeeding intention, initiation, any breastfeeding at 6 months, and 6 month exclusive breastfeeding with inadequate prenatal care and other potential predictors (Cont'd)

Independent Variables	Intended to Breastfeed	Initiated Breastfeeding	Terminated Breastfeeding at 6 months	Exclusive Breastfeeding at 6-months
	Adjusted OR (95%CI)†	Adjusted OR(95%CI)†	Adjusted OR (95%CI)†	Adjusted OR (95%CI)†
Pregnancy-Related Characteristics				
**Gravidity**				
Primigravida	**1.54 (1.33-1.79)**	**1.64 (1.33-2.03)**	**1.18 (1.04-2.56)**	**1.19 (1.02-2.68)**
Multigravida	1.00	1.00	1.00	1.00
**Reaction to pregnancy**				
Happy	1.08 (0.64-1.82)	0.78 (0.38-1.58)	0.87 (0.51-1.50)	0.85 (0.49-1.47)
Indifferent	0.92 (0.62-1.37)	0.76 (0.43-1.32)	1.12 (0.73-1.73)	0.95 (0.47-1.93)
Unhappy	1.00	1.00	1.00	1.00
**Health problems during pregnancy**				
No	1.16(0.98-1.37)	1.22(0.98-1.52)	1.08 (0.93-1.28)	1.14 (0.91-1.42)
Yes	1.00	1.00	1.00	1.00
**Cigarette smoking during pregnancy**				
No	**2.07 (1.66-2.58)**	**2.53 (1.94-3.30)**	**2.52 (1.95-3.26)**	**2.76 (1.71-4.44)**
Yes	1.00	1.00	1.00	1.00
Delivery Characteristics				
**Type of PNC provider**				
Obs/Gyn	1.00	1.00	1.00	1.00
Family Doctor	**1.20 (1.04-1.39)**	**1.47 (1.21-1.80)**	**1.31 (1.12-1.54)**	**1.26 (1.02-1.54)**
Midwife	**2.60(1.69-4.03)**	**3.60 (1.61-7.87)**	**2.28 (1.57-3.32)**	**1.91 (1.31-2.78)**
Nurse or nurse practitioner/other	1.10(0.71-2.52)	1.10(0.71-2.52)	1.16(0.71-2.52)	0.99(0.71-2.52)
**Type of Delivery**				
Vaginal	**1.27 (1.09-1.47)**	1.14 (0.92-1.42)	1.18 (1.00-1.39)	1.13 (0.91-1.40)
Cesarean	1.00	1.00	1.00	1.00
**Birth Setting**				
Hospital	1.00	1.00	1.00	1.00
Birthing center/Private home	1.44 (0.94-11.23)	2.35 (0.91-12.88)	**2.83 (1.17-6.83)**	**2.41 (1.34-4.03)**
Postpartum Characteristics				
**Baby's admission to the NICU**				
No	**1.23 (1.03-1.47)**	**1.67 (1.31-2.13)**	**1.59 (1.30-1.95)**	**1.32 (1.30-1.95)**
Yes	1.00	1.00	1.00	1.00
**Work after delivery**				
No	1.13 (0.94-1.37)	1.29 (0.99-1.66)	**1.30 (1.07-1.58)**	**1.40 (1.07-1.58)**
Yes	1.00	1.00	1.00	1.00
**Support After delivery**				
All of the time /Most of the time	1.02 (0.73-1.40)	1.09 (0.70-1.70)	0.88 (0.60-1.32)	0.62 (0.37-1.02)
Some of the time	0.74 (0.51-1.08)	0.99 (0.58-1.67)	0.99 (0.64-1.24)	0.69 (0.46-1.05)
None /Little of the time	1.00	1.00	1.00	1.00
**Intimate Partner Violence**				
No	0.83 (0.66-1.04)	0.72 (0.52-1.01)	1.15 (0.92-1.45)	1.23 (0.86-1.77)
Yes	1.00	1.00	1.00	1.00

†CI-Confidence Interval
